# Systematic review of factors influencing length of stay in ICU after adult cardiac surgery

**DOI:** 10.1186/s12913-016-1591-3

**Published:** 2016-07-29

**Authors:** Ahmed Almashrafi, Mustafa Elmontsri, Paul Aylin

**Affiliations:** Department of Primary Care and Public Health, School of Public Health, Imperial College London, Charing Cross Campus, Reynolds Building, St Dunstans Road, London, W6 8RP UK

**Keywords:** Cardiac ICU resource utilisation, Length of stay, Cardiac surgery

## Abstract

**Background:**

Intensive care unit (ICU) care is associated with costly and often scarce resources. In many parts of the world, ICUs are being perceived as major bottlenecks limiting downstream services such as operating theatres. There are many clinical, surgical and contextual factors that influence length of stay. Knowing these factors can facilitate resource planning. However, the extent at which this knowledge is put into practice remains unclear. The aim of this systematic review was to identify factors that impact the duration of ICU stay after cardiac surgery and to explore evidence on the link between understanding these factors and patient and resource management.

**Methods:**

We conducted electronic searches of Embase, PubMed, ISI Web of Knowledge, Medline and Google Scholar, and reference lists for eligible studies.

**Results:**

Twenty-nine papers fulfilled inclusion criteria. We recognised two types of objectives for identifying influential factors of ICU length of stay (LOS) among the reviewed studies. These were general descriptions of predictors and prediction of prolonged ICU stay through statistical models. Among studies with prediction models, only two studies have reported their implementation. Factors most commonly associated with increased ICU LOS included increased age, atrial fibrillation/ arrhythmia, chronic obstructive pulmonary disease (COPD), low ejection fraction, renal failure/ dysfunction and non-elective surgery status.

**Conclusion:**

Cardiac ICUs are major bottlenecks in many hospitals around the world. Efforts to optimise resources should be linked to patient and surgical characteristics. More research is needed to integrate patient and surgical factors into ICU resource planning.

## Background

Cardiac Intensive Care units (ICU) are specialised units that provide care to patients after cardiac surgery or those who are critically ill. Care provided in ICUs is costly and labour intensive. This is also coupled with limited number of ICU beds leading most ICUs to operate near full capacity [1]. Thus, unavailability of beds become an issue and may substantially impact upon other services such as operating theatres. Simply extending ICU capacity may not be feasible, due to physical limitations, resources or government regulation [2].

Patients receiving cardiac care are a heterogeneous group in their use of resources. The wide variation in length of stay (LOS), for example, is influenced by several clinical and non-clinical factors. This is also exacerbated by the complexity and the invasive nature of heart surgery. Most patients, depending on the hospital setting, are expected to be admitted to an ICU after their surgery. Standard care will be provided that include continuous ECG monitoring, hemodynamic management, pain control, renal monitoring, ventilation support and respiratory management [3, 4]. Thus, ICU stay is an important milestone in the cardiac patient hospital journey.

Several studies attempted to identify the effect of patients, treatment and institutional factors that can best explain variation around ICU LOS. However, there is still ambiguity and lack of concise recommendations on how resource planning can be improved by monitoring variation. Understanding variability could allow healthcare resource planners to allocate patients or resources in a way that maximises patient throughputs. However, integrating patient and treatment-related factors into the resource planning process is an area that still needs to be addressed. In general, there is a scarcity of literature about understanding how factors affecting ICU LOS can be translated into practice to optimise ICU resources.

While many studies [5–9] have identified factors associated with prolonged ICU LOS for patients undergoing cardiac surgery, the conclusions reached have not been reviewed as to how they can be of practical use to patients or resource management. Among several options available to clinicians and hospital managers are: targeting specific modifiable risk factors, expected LOS-based scheduling, capacity management, fast track anaesthesia, staffing levels and other resource planning strategies. This review aims to provide recommendations on how to approach this gap.

### Objectives

To systematically review the available literature in order to identify factors associated with LOS in ICU following cardiac surgery and to explore the evidence on the link between understanding these factors and patient and resource management. We provide recommendations on how these factors can be incorporated into decisions to improve resource utilisation.

### Questions

Our systematic review was driven by the following two questions: 1) what type of factors influence cardiac ICU length of stay? 2) Do the selected studies explore any application (i.e. medical or administrative) to improve cardiac ICU resource allocation based on an understanding of these factors?

## Methods

The systematic review was conducted and reported in accordance with the Preferred Reporting Items for Systematic Reviews and Meta Analyses Statement (PRISMA).

### Search strategy

Electronic searches of Embase, PubMed, ISI Web of Knowledge, Medline and Google Scholar were conducted using the following free text terms: prolonged length of stay OR long stay OR excess, intensive care unit OR critical care, determinants OR predictors OR risk factors OR factors, cardiac OR heart. Terms are summarised in Table 1. References contained in the included studies were checked for additional papers that were not identified in the electronic search.

**Table 1:** Search term used in electronic database

	Search terms
LOS	LOS, Extended LOS, long LOS, prolonged LOS, excess LOS
Surgery	Cardiac surgery, heart surgery, AND post*
Intensive care unit	Critical care, cardiac ICU, ICU, and intensive care
Management strategies	Resource planning, bed management, patient flow, scheduling, throughputs, and efficiency

*used to find other derivates associated with theterm

#### Study criteria

Abstracts were examined by two reviewers (AA and ME) and were selected or excluded based on the following criteria:

### Selection criteria

Studies were included if they met the following criteria: 1) reported association between variables of interest and postsurgical LOS for adult patients who underwent cardiac surgeries only, 2) were published between January 2005 and January 2015 in English language and in peer-reviewed journals. We restricted search to this time period to account for advances both in treatment and medical technology. It is more likely that factors affecting LOS have changed over time due to reduction of severity of several risk factors influential to LOS. Recent advancements in perioperative care may have also contributed to this change. Therefore, we believe a period of 10 years is a reasonable time to reflect these changes, and 3) we also included studies with main goal of evaluating ICU LOS predictive models since these models were derived from statistically significant factors influential to LOS.

### Exclusion criteria

Studies were excluded if they 1) had no reference to length of stay as a measure of outcome (e.g. studies reporting costs only), 2) studies that have investigated resource utilisation among medical patients (e.g. heart failure) and have not included patients who underwent a cardiac surgery, and 3) studies that have exclusively investigated factors affecting the general postoperative LOS without a reference to factors affecting LOS in the intensive care setting.

#### Data extraction and quality assessment

Using a standardised data collection form, we extracted data from the selected studies relating to design, patient sample size, identified significant factors, type of surgery, statistical method used and number of hospitals in the study. We also reviewed any reported recommendations on resource or patient management interventions. We define management intervention in this context as any strategy geared toward improving patient scheduling, reducing LOS, improving patient flow or resource allocation in general, utilising knowledge on factors affecting LOS.

### Quality assessment

A quality assessment of papers was conducted using an adapted version of the Newcastle-Ottawa Scale (NOS) [10]. The NOS uses a star rating system to judge the quality of a study. We assessed each article for adhering to the following criteria:

### Selection

1) Representativeness of the sample: a) the sample is truly representative of patients who underwent cardiac surgery (all subjects or random sampling) (one star), b) the sample is somewhat representative (non-random sampling) (one star), and c) selected group of users (no star), d) no description of the sampling strategy (no star). 2) Sample size: a) justified and satisfactory (one star), and b) not justified (no star). Maximum 3 stars.

### Comparability

1) confounding factors: a) the study controls for the most important factor (one star), and b) the study control for any additional factor (one star). Maximum 2 stars.

### Outcome

1) assessment of the outcome: a) independent blind assessment (two stars), b) record linkage (two starts), c) self-report (one star), and d) no description (no star). 2) statistical test: a) the statistical test used to analyse the data is clearly described and appropriate, and the measurement of the association is presented, including confidence intervals and probability level (*p* value) (one star), and b) the statistical test is not appropriate, not described or incomplete (no star). Maximum 6 stars.

We computed the total scores based on the assessment. The total possible stars is 11. The assessment can be found in Appendix B.

### Data synthesis

It was not possible to combine all result and conduct a meta-analysis due to substantial methodological and clinical heterogeneity of the studies.

## Results

**Fig. 1:**
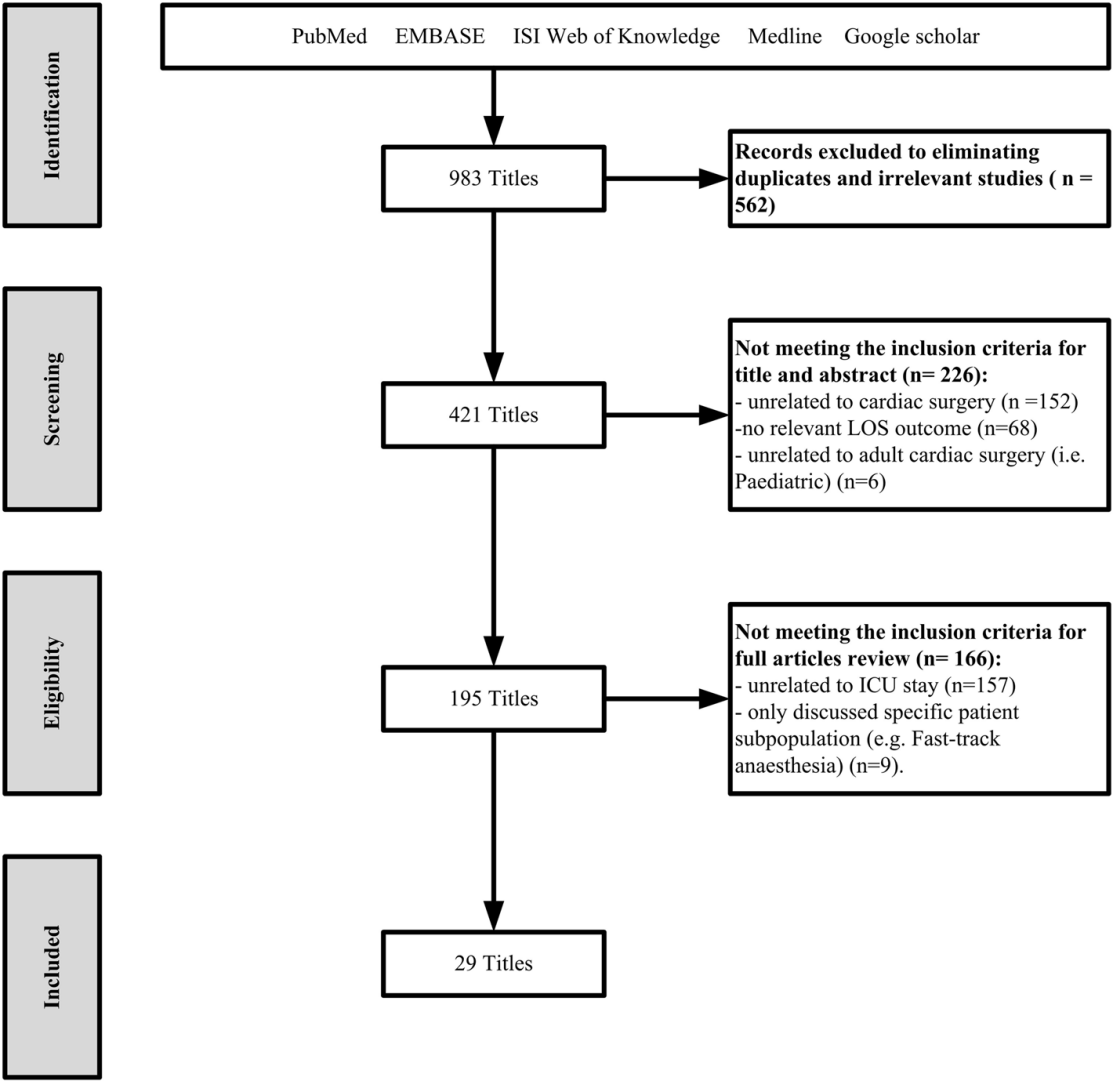
Search process flow for articles included in the review

We identified 983 papers in the initial search. Papers were then reviewed for relevancy based on their titles or abstracts. Eventually, 29 papers met the inclusion criteria (Fig. 1). Of these papers 27 were cross-sectional, and 2 were case-control. The majority of the selected papers 22 (76 %) were conducted in developed countries. Several studies have specifically addressed a single LOS predictor such as advanced age [11], blood transfusion [12], surgical wound infection [13], hypoactive delirium [14], or serum creatinine [15]. 21 (72 %) assessed LOS in relationship to several preoperative, intraoperative or postoperative variables rather than limiting analysis to one stage of hospital stay. No study has collected data from more than a single institution. A summary of the selected studies is provided in Appendix A.

Multivariate logistic regression was commonly used as a statistical tool in 23 papers. Other statistical analysis carried out were proportional hazard [16], survival analysis [17], case-control [13], regression tree [18], or combination of statistical hypothesis tests.

### Prolonged LOS: lack of uniformed definition

The majority of the studies have dichotomised their LOS into two groups (normal vs. prolonged LOS), hence the use of the logistic regression models. Only four studies have treated LOS as a continuous variable (Appendix A). This might have been the case because LOS data are highly skewed and subject to outliers precluding modelling LOS as normally distributed variable [19]. In addition, it is more meaningful to separate patients into two groups since patients with prolonged LOS are an important subpopulation that impact use of hospital resources. However, we further observed a variation in the selection of the cut-off points that define prolonged LOS. These ranged from 24 h to 7 days.

### The overall objective of identifying predictors of ICU LOS

We categorised studies based on their objectives for identifying predictors of LOS. Two types emerged from our review: 1) general descriptions of predictors (22 papers), and 2) risk prediction for prolonged ICU LOS (7papers). Most studies fell into the first category where predictors are selected among several variables. Studies in the second category attempted to derive predictive models that stratified patients based on their risk for prolonged LOS which potentially can be used to facilitate the selection of optimal patient management strategy. Studies with prediction models had on average 10 predictors which were derived from hospital routinely collected data.

The majority of the reviewed papers didn’t report specific real-world application that might be realised from understanding influential predictors. However, two studies reported implementation [20, 21]. With such a small rate, it is difficult to assess the acceptance and usefulness of these studies in improving operational performance of ICU units.

### Independent predictors of ICU LOS

Several studies included factors that are likely to exist early during a hospitalisation. This is especially the case when the aim was designing a predictive model. However, definitions of variables were not commonly discussed with the exception of Augoustides et al., [22] De Cocker et al., [16] Rosenfeld et al., [23] and Widyastuti et al. [24] who reported definitions of variables used in assessment.

Most studies included basic demographic variables such as gender, age, and race. We identified patient fixed variables (e.g. gender, body mass index) to be the most commonly studied variables. Yet, few turned to be independently significant when a multivariate analysis was used. Age was the most commonly reported statistically significant predictor. Only two studies found gender to be a contributing factor of prolonged ICU LOS. Conversely, BMI was independently significant in only two studies and body service area was not independently significant in any of the reviewed studies. As shown in Table 2, comorbidities accounted for a large proportion of risk for prolonged LOS. This is because several studies were designed to predict LOS at time of admission.

**Table 2:** Predictors of ICU LOS after cardiac surgery

Predictive factors	Reference	Predictive factors	Reference
Patient related factors		Arrhythmia / Atrial fibrillation	[5, 9, 16, 20, 21, 28, 34, 35]
Increased age	[11, 23, 24, 28, 34, 36] [5, 8, 16, 18, 20, 26]	Low Ejection Fraction	[21, 34, 36] [20, 22, 29]
Gender	[6, 16]	Left ventricular dysfunction	[17, 37]
BMI	[20, 38]	NYHA class III-IV	[6, 16, 25, 34, 36]
Body service area	None	Chronic heart failure	[11, 21, 24]
Smoking	[20, 39]	Critical preoperative state	[34]
Platelet count	[28]	Hypoactive delirium	[14]
hyperglycaemia	[35]	Surgical factors	
High preoperative serum creatinine	[15, 17, 35]	Non-elective surgery	[16, 17, 20, 23–25, 29, 36, 37]
Fat-free mass index	[40]	Type of surgery	[14, 16]
Plasma disappearance rate of indocyanine green	[18]	CPB use	[20]
Previous cardiac surgery	[5, 24, 36, 37]	Bypass time	[23–25]
Comorbidities		Cross clamp time	[38]
Hypertension	[5, 20, 23]	Combined surgery	[34]
COPD	[11, 21, 23, 24, 34, 36]	Intra-aortic balloon pump	[16, 24]
Diabetes	[20, 39]	Blood transfusion	[7, 12, 24]
Insulin-dependent diabetes	[21]	Inotropes support	[7, 16, 25, 28]
Hypercholesterolemia	[20]	Triple vessel or left main disease	[36]
Recent Myocardial infarction	[36]	Complications	
Renal failure/ dysfunction	[11, 21, 22, 29, 36, 41]	Reoperation for bleeding	[26]
Unstable Angina	[37]	Pulmonary	None
Pulmonary hypertension	[37]	Cardiac	None
Angina class IV	[20]	Neurological	[22]
Peripheral vascular disease	[20, 24, 36]	Infection	[13]
		Renal complications	None

*Abbreviation*: *COPD* chronic obstructive pulmonary disease, *BMI* Body Mass Index, *CPB* Cardiopulmonary bypass machine

Surgical characteristics such as the use of Cardiopulmonary Bypass (CPB) machine [20], bypass time [23–25] and blood transfusion [7, 12, 24] were predictive of ICU LOS. Postoperative complications were less commonly discussed. There were only 4 studies [13, 22, 26] that carried out analysis with some postoperative complications included.

We observed some consistency over some factors that have been found to be independently associated with patient stay. For example, the following variables were identified to be independent predictors of ICU LOS in four or more studies (Table 2): increased age, Chronic Obstructive Pulmonary Disease (COPD), renal failure or dysfunction, atrial fibrillation, low ejection fraction, NYHA class III-IV, non-elective surgery, previous cardiac surgery, and inotropes support.

Unlike other studies which neglected the inclusion of patients who died before they were discharged from the ICU, De Cocker et al. utilised Cox Proportional Hazards model which accounted for these patients in the analysis [16]. The rationale for including them was that most of these patients would probably have had extended ICU stays if they stayed alive. Ghotkar and colleagues conducted a sensitivity analysis whereby patients who died were included in a second analysis [20]. The independent risk factors identified originally remained unchanged. Barili et al. [17] developed a model to identify predictors of ICU LOS in patients who were discharged a live from ICU and another model for those patients who died.

## Discussion

### Variation in definitions

Our findings are similar to those reported by Messaoudi et al. who pointed out to the fact that there was a lack of uniformed and standardized definitions regarding ICU length of stay [27]. In most of our reviewed studies, the cut-off criteria was more likely to be arbitrary. Only a single study reported a definition that was based on a clinical consideration. For example, Rosenfeld et al. selected 7 days as a cut-off point because it may indicate a severe complications [23]. The choice of the threshold periods may also be determined by the average stay duration in a particular ICU unit. For instance, when step down units are available, patients can be transferred to these units to free up some ICU beds. In such facilities, patients, on average, will have a shorter ICU LOS.

Few studies have provided definitions of the variables. However, even when the definitions of variables are provided, it is possible that they varied across surgeons even in the same hospital [5]. This might be a common weakness in some of our reviewed studies where data were retrospectively collected.

### Type of surgery

Thirteen studies have included only a single type of surgery in their analysis. Out of these studies, 9 included isolated Coronary Artery Bypass Grafting (CABG) surgery only. However, cardiac ICU beds are shared by all types of cardiac surgical patients. From a resource allocation perspective, these patients compete for the same resources and thus disregarding certain patient types will undermine the usefulness of these studies in resource management.

### The utility of understanding influential factors of ICU LOS

The majority of the studies have identified factors predicting LOS without reference to a particular use in operational or clinical application. Understanding patient variability around resource consumption is an important task that should be undertaken by hospital managers. Continuous surveillance of factors affecting cardiac ICU LOS may allow better design of services and streamline patients more efficiently. However, there is paucity in literature on whether hospitals are integrating these risk factors into resource planning. As stated previously, the majority of the reviewed studies have not demonstrated the applicability of their findings in improving the clinical or operational performance.

Factors contributing to patient ICU LOS variability can be potentially integrated into resource management practices. Broadly speaking, the utility of such knowledge can be applicable to patient management (e.g. aggressive treatment of comorbidities, fast track triage) and resource management (e.g. scheduling surgery, bed allocations, or determining staffing level).

An early recognition of patients at risk of prolonged LOS was the main objective of the prediction models. Our review revealed that these models varied widely on the types of variables included. In addition, all of these models were based on single institution which casts a doubt on their generalisability beyond the local setting where they have been originally derived from. This supports our hypothesis that the practice of ICU LOS prediction is still locally based. Clearly, there is a need for a simple [21] and universal model that takes into consideration differences in patient and surgical characteristics. However, Widyastui et al. argued that a universal model may also be difficult to develop as LOS distributions depend on institutional policies governing ICU discharge [24]. It is worth mentioning that the utility of ICU LOS prediction models in improving patients and resource management has not been investigated in the literature. We aim to address this in a future study.

Based on our analysis of the studies, two options can be pursued when analysing factors predictive of ICU LOS. The first is investigate these factors using available data that are usually routinely collected in most hospitals. Second, there might be a need to evaluate a single common factor (e.g. atrial fibrillation that might be prevalent among the local population) to assess its impact on resource use.

Segmenting patients based on their expected LOS should augment decision making for resource allocation in surgical care. For example, Wagener et al. developed a prediction model that can discriminate between patients who are candidates of fast-track protocol [21]. The goal of instituting a fast track protocol was to minimise the time patients remain in the ICU or bypass it altogether. They further noted that the existing tools were not reliable to identify patients for fast-track protocols and their model was superior to these systems. Another application was suggested by Tribuddharat and colleagues in the form of a prediction tool that can identify patients with prolonged LOS and may provide the anaesthesiologist with enough time to correct risk factors [28]. Targeting risk factors through aggressive treatment regimens prior to surgery may reduce the proportion of patients who require lengthy ICU LOS which can result in several medical, operational and financial benefits. This is the case because many of the risk factors are potentially modifiable. Consequently, aggressive preoperative treatments and workups prior to surgery can mitigate the need for extended LOS [29]. Scheduling patients based on their expected LOS is another way of expanding the applicability of early recognition of factors influential to ICU LOS. For example, patients expected to stay longer in the ICU can be scheduled at the end of the week to take advantage of weekends when no surgical procedures are scheduled [30].

### Variable selections

Most studies utilised routinely collected data available in most hospitals information systems. These databases include demographic, comorbidities, complications, and surgery detail. Such data are readily available. However, there are several contextual factors that affect ICU LOS that are often overlooked. For example, in hospitals that practice a protocol of fast tracking, the normal duration of stay post cardiac surgery will be less than for hospital that don’t implement this system. It will also depend on whether patients can be transferred to a step-down unit which is less resource-intensive than conventional ICUs. Moreover, LOS distributions were found to depend on institutional policies which greatly influence discharge practices [24]. For example, individual physicians’ judgement is a factor that is associated with prolonged LOS in the ICU [27, 31]. Additionally, as Cots et al. suggest, the size of the hospital can affect LOS. As such, large urban teaching hospitals had more patients with very high LOS compared to medium and small community hospital [32].

Difference in LOS variability can also be explained by the capacity of the ICU, the level of demand for ICU beds, and the surgeon skills. Therefore, the decision to discharge patients from ICU is mostly based on policy as well as medical criteria [24].

Even though we observed that all reviewed studies have not included variables related to hospital or social settings (i.e. contextual factors), we believe that the hospital routinely collected data are adequate in identifying factors affecting LOS and therefore can be used in assessing resource utilisation in hospital systems. Similarly, for a wider practical application, administrative data can be utilised to describe factors contributing to resource use [33]. This provides an opportunity to consider data commonly collected in most hospitals and improve generalisability of models.

When lack of data is an issue or when the purpose is to predict ICU LOS, researchers should aim for a parsimonious model that explains the largest variation with as few predictors as possible. Equally important, some of our reviewed studies had a sample size that is relatively small (e.g. < 200). With such size, it is possible that these studies missed the inclusion of rare events such as renal failure and stroke which have high impact on ICU LOS. Moreover, in some statistical analysis, low sample size precludes including complete list of potential factors affecting LOS and risk reducing the power of the study.

## Conclusion

Patient and surgical factors are valuable information for predicting LOS in critical care. However, the extent at which these factors are utilised in managing patients is unclear. Studies vary on the type of predictors being selected. Few variables were more common than others. For example, atrial fibrillation/ arrhythmia, increased age, chronic obstructive pulmonary disease (COPD), renal failure/ dysfunction and non-elective surgery status were common predictors.

Identifying risk factors for prolonged LOS should not be treated in isolation of the intended use. That is, the utility of identifying risk factors should be clearly defined. This will facilitate the integration of influential factors into the resource allocation decision making process. This may also allow hospital stakeholders (e.g. bed managers) to engage in case-mix evaluation and thus empirically assess resource needs. More research is needed to link variation around hospital resource use and management strategies designed to optimise patients flow.

The cut-off period for prolonged LOS should be carefully selected taking into consideration clinical judgement as well as past historical data. It might be more accurate to assess LOS as a continuous variable using appropriate statistical methods to adjust for different variables. This will potentially eliminate the arbitrary selection of an endpoint. Similarly, studies examining resource utilisation should clearly define variables as differences in definitions can substantially affect results.

Unique local practices should not be underestimated when investigating factors influencing hospital resource utilisation. Several organisational contexts can impact LOS. However, we acknowledge that it is difficult to account for organisational factors due to the difficultly associated with measuring some of these factors.

Equally, the unique characteristics of patients treated by individual hospitals adds another challenge in predicting LOS across multiple settings. Our review can inform researchers interested in this evaluation to focus on those variables that are commonly reported to be independent contributors to ICU LOS (see Table 2).

### Limitations

We only reviewed studies published in the English language. This means that studies written in other languages which may meet the inclusion criteria were excluded.

## Abbreviations

BMI, body mass index; CABG, coronary artery bypass grafting; COPD, chronic obstructive pulmonary disease; CPB, cardiopulmonary bypass machine; ICU, Intensive care unit; LOS, length of stay.

## Appendix A

**Table 3:** Studies summary

RN	First author and year	Country	Number of hospitals	Total patients	Cardiac surgery type	LOS definition	Objective type
1	Wang, 2012 [34]	China	1	3925	Valve surgery	Prolonged > 7 days	Prediction model
2	Atoui, 2008 [29]	Canada	1	426	CABG, valve, and combined surgery	Prolonged ≥ 2 days	General description of predictors
3	Abrahamyan 2006 [5]	Armenia	1	391	Isolated CABG	Prolonged ≥ 3 days	General description of predictors
4	Augoustides 2006 [22]	USA	1	144	Thoracic Aortic surgery	Prolonged > 5 days	General description of predictors
5	Ghotkar 2006 [20]	UK		5186	Isolated CABG	Prolonged > 3 days	Prediction model
6	Hein 2006 [26]	Germany	1	2683	CABG, combined, and other	Prolonged > 3 days	General description of predictors
7	Rosenfeld 2006 [23]	USA	1	9869	Isolated CABG	Prolonged ≥ 7 days	General description of predictors
8	Horer 2008 [9]	Germany	1	281	Atrial septal defect closure	Prolonged > 2 days	General description of predictors
9	Lei 2009 [25]	China	1	298	Aortic arch surgery	Prolonged > 5 days	General description of predictors
10	Widyastuti 2012 [24]	Norway	1	4994	CABG, Valve, Combined, and other	Prolonged > 2, 5, 7 days	General description of predictors
11	Wagener 2011 [21]	USA	1	1201	CABG, valve, Combined, and other	ICU LOS < 2 days ICU LOS > 7 days	Prediction model
12	Tribuddharat 2014 [28]	Thailand	1	168	CABG, Valve, and combined	ICU LOS < 42 h	Prediction model
13	Scott 2005 [11]	USA	1	1746	CABG	Continuous	General description of predictors
14	Scott 2008 [12]	USA	1	1746	CABG	Continuous	General description of predictors
15	Herman, 2009 [36]	Canada	1	3483	CABG	Prolonged > 3 days	Prediction model
16	Graf, 2010 [13]	Germany	1		CABG	Continuous	General description of predictors
17	Giakoumidakis, 2011 [41]	Greece	1	313	CABG, valve, and combined	Prolonged ≥ 2 days	General description of predictors
18	De Cocker, 2011 [16]	Belgium	1	1566	CABG, valve, combined, and other	Prolonged > 2, 5, 7 days & continuous	Prediction model
19	Cacciatore, 2012 [6]	Italy	1	250 (≥65 years)	CABG, valve, and combined	Prolonged ≥ 3 days	General description of predictors
20	Azarfarin, 2014 [7]	Iran	1	280	CABG, valve, and combined	Prolonged > 4 days	General description of predictors
21	Nakasuji, 2005 [8]	Japan	1	100	CABG	Prolonged > 3 days	General description of predictors
22	Eltheni, 2012 [35]	Greece	1	150	CABG, valve, combined, and other	Prolonged ≥ 2 days	General description of predictors
23	Bignami, 2012 [38]	Italy	1	27	CABG, valve, combined, other	Prolonged > 24 h	General description of predictors
24	Stransky, 2011 [14]	Germany	1	506	CABG, valve, combined, other	Continuous	General description of predictors
25	Weis, 2014 [18]	Germany	1	190	CABG, valve, combined	Prolonged > 3 days	General description of predictors
26	Ezeldin, 2013 [15]	Saudi Arabia	1	1033	CABG, valve	Prolonged > 3 days	General description of predictors
27	Oliveira, 2013 [39]	Brazil	1	104	CABG	Prolonged > 3 days	General description of predictors
28	Venrooij, 2011 [40]	Netherlands	1	325	CABG, valve, combined	Not specified	General description of predictors
29	Barili	Italy	1	3861	CABG, valve, combined, other	Continuous	Prediction model

## Appendix B

**Table 4:** Newcastle-Ottawa modified for cross-sectional studies

Study	Selection						Comparability		Outcome						Total
	Representativeness of the sample				Sample size		Confounding factors		Assessment				Statistical test		
	A*	B*	C	D	A*	B	A*	B*	A**	B**	C*	D	A*	B	
1. Abrahamyan, L., et al., Determinants of Morbidity and Intensive Care Unit Stay after Coronary Surgery. Asian Cardiovascular and Thoracic Annals		+			+					+			+		5
2. Atoui, R., et al., Risk factors for prolonged stay in the intensive care unit and on the ward after cardiac surgery.		+			+		+	+					+		4
3. Augoustides, J.G., et al., Clinical predictors for prolonged intensive care unit stay in adults undergoing thoracic aortic surgery requiring deep hypothermic circulatory arrest.		+			+		+			+			+		5
4. Azarfarin, R., et al., Factors influencing prolonged ICU stay after open heart surgery.					+		+			+			+		5
5. Cacciatore, F., et al., Determinants of prolonged intensive care unit stay after cardiac surgery in the elderly.	+				+		+	+		+			+		7
6. De Cocker, J., et al., Preoperative prediction of intensive care unit stay following cardiac surgery.	+				+		+	+		+			+		7
7. Eltheni, R., et al., Predictors of prolonged stay in the intensive care unit following cardiac surgery.	+				+		+			+			+		6
8. Ghotkar, S.V., et al., Preoperative calculation of risk for prolonged intensive care unit stay following coronary artery bypass grafting.	+				+					+			+		5
9. Giakoumidakis, K., et al., Risk factors for prolonged stay in cardiac surgery intensive care units.		+			+				+		+		+		6
10. Hein, O.V., et al., Prolonged intensive care unit stay in cardiac surgery: risk factors and long-term-survival.	+				+		+			+			+		6
11. Herman, C., et al., Predicting prolonged intensive care unit length of stay in patients undergoing coronary artery bypass surgery--development of an entirely preoperative scorecard.	+				+		+			+			+		6
12. Horer, J., et al., Risk factors for prolonged intensive care treatment following atrial septal defect closure in adults.		+				+				+				+	3
13. Lei, Q., et al., Preoperative and intraoperative risk factors for prolonged intensive care unit stay after aortic arch surgery.		+				+	+			+			+		5
14. Nakasuji, M., M. Matsushita, and A. Asada, Risk factors for prolonged ICU stay in patients following coronary artery bypass grafting with a long duration of cardiopulmonary bypass.	+					+				+			+		4
15. Scott, B.H., F.C. Seifert, and R. Grimson, Blood transfusion is associated with increased resource utilisation, morbidity and mortality in cardiac surgery.		+			+				+					+	5
16. Scott, B.H., et al., Octogenarians undergoing coronary artery bypass graft surgery: resource utilization, postoperative mortality, and morbidity.		+			+		+			+			+		6
17. Tribuddharat, S., et al., Development of an open-heart intraoperative risk scoring model for predicting a prolonged intensive care unit stay.		+				+	+			+			+		5
18. Wagener, G., et al., The Surgical Procedure Assessment (SPA) score predicts intensive care unit length of stay after cardiac surgery.		+			+		+			+			+		5
19. Wang, C., et al., Risk model of prolonged intensive care unit stay in Chinese patients undergoing heart valve surgery.	+				+		+			+			+		6
20. Widyastuti, Y., et al., Length of intensive care unit stay following cardiac surgery: is it impossible to find a universal prediction model?	+				+		+			+			+		6
21. Bignami et al. Urinary neutrophil gelatinase-associated lipocalin as an early predictor of prolonged intensive care unit stay after cardiac surgery		+			+					+			+		5
22. Venrooij et al. The impact of low preoperative fat-free body mass on infections and length of stay after cardiac surgery: A prospective cohort study	+				+		+			+			+		6
23. Stransky et al. Hypoactive Delirium After Cardiac Surgery as an Independent Risk Factor for Prolonged Mechanical Ventilation	+				+		+			+			+		6
24. Ezeldin Relation between serum creatinine and postoperative results of open-heart surgery	+				+		+			+			+		6
25. Wies et al. Indocyanine green clearance as an outcome prediction tool in cardiac surgery: A prospective study	+				+		+	+		+			+		7
26. Barili et al. An original model to predict Intensive Care Unit length-of stay after cardiac surgery in a competing risk framework	+				+		+			+			+		6
27. Oliveira E, et al., Risk factors for prolonged hospital stay after isolated coronary artery bypass grafting	+				+		+			+			+		6

The number of stars for each item is represented by asterisks*

**Table 5:** Newcastle-Ottawa: case-control studies

Study	Selection										Comparability		Exposure										Total
	Case definition			Representativeness of the cases		Selection of controls			Definition of controls		Confounding factors		Ascertainment of exposure					Same method of ascertainment for case and controls		Non-response rate			
	A*	B	C	A*	B	A*	B	C	A*	B	A*	B*	A*	B	C	D	E	A*	B	A*	B	C	
28. Graf, K., et al., Economic aspects of deep sternal wound infections.		+		+			+		+		+		+					+			+		5
29. Rosenfeld, R., et al., Predictors and outcomes of extended intensive care unit length of stay in patients undergoing coronary artery bypass graft surgery.		+		+			+		+		+		+					+			+		5

The number of stars for each item is represented by asterisks*
